# Impact of radiologically stratified exacerbations: insights into pneumonia aetiology in COPD

**DOI:** 10.1186/s12931-018-0842-8

**Published:** 2018-07-28

**Authors:** Nicholas P. Williams, Kristoffer Ostridge, Jeanne-Marie Devaster, Viktoriya Kim, Ngaire A. Coombs, Simon Bourne, Stuart C. Clarke, Stephen Harden, Ausami Abbas, Emmanuel Aris, Christophe Lambert, Andrew Tuck, Anthony Williams, Stephen Wootton, Karl J. Staples, Tom M. A. Wilkinson, on behalf of the AERIS Study Group, J. Alnajar, R. Anderson, E. Aris, W. R. Ballou, A. Barton, S. Bourne, M. Caubet, S. C. Clarke, D. Cleary, C. Cohet, N. A. Coombs, K. Cox, J.-M. Devaster, V. Devine, N. Devos, E. Dineen, T. Elliot, R. Gladstone, S. Harden, J. Jefferies, V. Kim, C. Lambert, S. Mesia-Vela, P. Moris, K. Ostridge, T. G. Pascal, M. Peeters, S. Schoonbroodt, K. J. Staples, A. Tuck, L. Welsh, V. Weynants, T. M. A. Wilkinson, A. P. Williams, N. P. Williams, C. Woelk, M. Wojtas, S. Wootton

**Affiliations:** 1 Clinical and Experimental Sciences, University of Southampton Faculty of Medicine, Southampton General Hospital, Southampton, UK; 2 Southampton NIHR Respiratory Biomedical Research Unit, Southampton General Hospital, Southampton, UK; 3 GSK Vaccines, Rixensart, Belgium; 4 Primary Care and Population Sciences, Faculty of Medicine, University of Southampton, Southampton, UK; 5 Wessex Investigational Sciences Hub, University of Southampton Faculty of Medicine, Southampton General Hospital, Southampton, UK; 6 Department of Radiology, University Hospital Southampton NHS Foundation Trust, Southampton General Hospital, Tremona Road, Southampton, UK; 7 Faculty of Medicine and Institute for Life Sciences, University of Southampton, Southampton, UK; 8 Southampton NIHR Biomedical Research Centre, University Hospital Southampton NHS Foundation Trust, Southampton, UK; 9 Present address: Portsmouth Hospitals NHS Trust, Queen Alexandra Hospital, Portsmouth, UK

**Keywords:** COPD, Pneumonia, Infiltrates, Exacerbations

## Abstract

**Background:**

COPD patients have increased risk of developing pneumonia, which is associated with poor outcomes. It can be symptomatically indistinguishable from exacerbations, making diagnosis challenging. Studies of pneumonia in COPD have focused on hospitalised patients and are not representative of the ambulant COPD population. Therefore, we sought to determine the incidence and aetiology of acute exacerbation events with evidence of pneumonic radiographic infiltrates in an outpatient COPD cohort.

**Methods:**

One hundred twenty-seven patients with moderate to very severe COPD aged 42–85 years underwent blood and sputum sampling over one year, at monthly stable visits and within 72 h of exacerbation symptom onset. 343 exacerbations with chest radiographs were included.

**Results:**

20.1% of exacerbations had pneumonic infiltrates. Presence of infiltrate was highly seasonal (Winter vs summer OR 3.056, *p* = 0.027). In paired analyses these exacerbation events had greater increases in systemic inflammation. Bacterial detection rate was higher in the pneumonic group, with *Haemophilus influenzae* the most common bacteria in both radiological groups. Viral detection and sputum microbiota did not differ with chest radiograph appearance.

**Conclusions:**

In an outpatient COPD cohort, pneumonic infiltrates at exacerbation were common, and associated with more intense inflammation. Bacterial pathogen detection and lung microbiota were not distinct, suggesting that exacerbations and pneumonia in COPD share common infectious triggers and represent a continuum of severity rather than distinct aetiological events.

**Trial registration:**

Trial registration Number: NCT01360398.

**Electronic supplementary material:**

The online version of this article (10.1186/s12931-018-0842-8) contains supplementary material, which is available to authorized users.

## Background

Acute exacerbations of COPD are associated with impaired quality of life, accelerated lung function decline and place considerable burden on health-care resources [1–3]. They are typically inflammatory events [4, 5], triggered by bacterial and viral infection [6], often requiring treatment with antibiotics and oral corticosteroids (OCS) either in an ambulatory setting, or less frequently in hospital [7]. As a result, they have become a key target for intervention in COPD. With this recognition, the emergence of symptom, pathogen and biomarker-guided strategies now offers scope to phenotype exacerbations, potentially allowing for tailored management approaches [8].

Pneumonia is more common in patients with COPD [9], and can be clinically indistinguishable from exacerbations. Numerous factors associated with pneumonia risk have been reported in COPD, including inhaled corticosteroid (ICS) use, comorbidity and nutritional status [10, 11]. COPD patients requiring hospitalisation for acute respiratory tract illness, often have complicating pneumonic infiltrate evident on chest radiograph (CXR) [12]. Moreover, these hospitalised episodes are associated with more intense inflammatory responses, differing pathogen profiles and often lead to longer hospital stays and increased rates of readmission [13–15]. This raises important treatment dilemmas, particularly around the use of OCS, risk-stratification for ICS and antibiotic usage. This is particularly relevant in the era of emerging antimicrobial resistance, where clinical strategies to better stratify and target antibiotics effectively are vital.

Validating a diagnosis of pneumonia requires the radiographic identification of pulmonary infiltrate in the context of symptoms of a respiratory tract infection which to date, has largely limited its study to hospitalised events. Given that only a comparatively small proportion of exacerbations require hospitalisation [16], our understanding of the frequency, nature and impact of pneumonic infiltrates complicating non-hospitalised exacerbations is currently lacking.

In a secondary analysis of the Acute Exacerbation and Respiratory InfectionS in COPD (AERIS) cohort, a prospective, longitudinal study of outpatients with COPD, we used radiological stratification to provide a detailed insight into the aetiology and character of exacerbations complicated by pneumonic change.

## Methods

### Study design and measurements

The AERIS study (ClinicalTrials.gov NCT01360398) was a prospective, observational cohort study conducted at the University Hospital Southampton, UK. The study protocol has been described previously [17]. Eligible patients with ≥1 treated exacerbation in the year prior to enrolment, and a confirmed diagnosis of COPD from a post-bronchodilator forced expiratory volume in 1 s (FEV1)/Forced vital capacity (FVC) ratio of < 0.7, were recruited. Patients on long-term antibiotic and/or OCS therapy were not eligible for inclusion. For full inclusion/exclusion criteria see Additional file 1. The study was conducted in accordance with the Declaration of Helsinki and Good Clinical Practice, and was approved by the Southampton and South West Hampshire Research Ethics Committee. All participants provided written informed consent.

Patients were followed monthly in the stable-state and reviewed within 72 h of exacerbation symptom onset. Exacerbations were detected using daily electronic diary cards. The definition and severity of exacerbations, has been previously described [6, 17]. All events were managed as an exacerbation from the outset, as judged appropriate by the clinical team, in accordance with local guidelines. Exacerbation onset date was used to describe seasonality with winter defined as December–February, spring as March–May, summer as June–August and autumn as September–November.

Posterior-anterior CXRs performed at acute visits, were reported by a blinded, designated thoracic radiologist (SH), with a randomly selected subset (*n* = 100) reported independently by a second, blinded thoracic radiologist (AA). Pneumonic infiltrates were characterised as acinar (lobar) or reticulo-nodular. Multiple exacerbations with pneumonic infiltrate in the same individual, were included if the infiltrate was new.

### Sample collection and analysis

Spontaneous or induced sputum samples were processed as previously described [6, 18, 19]. Sputum was cultured for bacteria and analysed by polymerase chain reaction (PCR) for bacterial and viral detection, monthly and at exacerbation (See Additional file 1: Supplementary methods). Blood analysis included differential white cell count, C-reactive protein (CRP), fibrinogen and procalcitonin (PCT) at study entry, exacerbation and at quarterly visits throughout the year.

We have extensively analysed the microbiome of the AERIS cohort and the in-depth analysis of correlations with disease severity and exacerbation phenotype have been previously reported [20]. In the current sub-analysis, we investigated whether there were differences in the abundance of specific phyla and genera in exacerbations with and without pneumonic infiltrate. For the microbiome analysis, genomic DNA was extracted from sputum using the MagNA Pure 96 DNA Volume Kit (Roche Diagnostics), according to the manufacturer’s instructions. The conserved V4 region of the 16S rRNA gene was amplified and sequenced on an Illumina MiSeq sequencer. Sequenced reads were filtered for quality and processed using the QIIME (quantitative insights into microbial ecology) pipeline version 1.8.0. Operational taxonomic units (OTUs) were subsequently clustered from chimera-cleaned reads at a 97% identity threshold and assigned taxonomy using the GreenGenes 16S RNA sequence database (version 13.8). For further methodological detail, see Additional file 1.

### Statistical analysis

Analyses were performed using SPSS version 22 (Armonk, NY:IBM Corp) and Stata version 14 (Stata Corporation, College Station, TX, USA). The sample size calculation for the AERIS study has been described previously [17]. Categorical data are presented as frequency, percentage (%) and were compared using the Chi-square test or Fisher’s exact test as appropriate. Continuous data are presented as mean, standard deviation (SD) if normally distributed and median, interquartile range (IQR) if not. Means were compared using the independent sample t-test and medians using the Mann-Whitney U test. For paired analyses comparing pre-exacerbation with exacerbation samples and stratifying by presence/absence of infiltrate, the Wilcoxon signed rank test was used. Clustered multivariate logistic regression, including the subject as a random effect, was used to account for individual patients having multiple exacerbations. Presence of pneumonic infiltrate at exacerbation was designated the dependent variable. Other factors judged by univariate analysis or, a priori to be of clinical importance were included as independent variables. *P*-values of < 0.05 were considered statistically significant. All analyses should be considered post hoc as they were not pre-specified in the AERIS statistical analysis plan.

## Results

### Patient characteristics and exacerbations

**Fig. 1:**
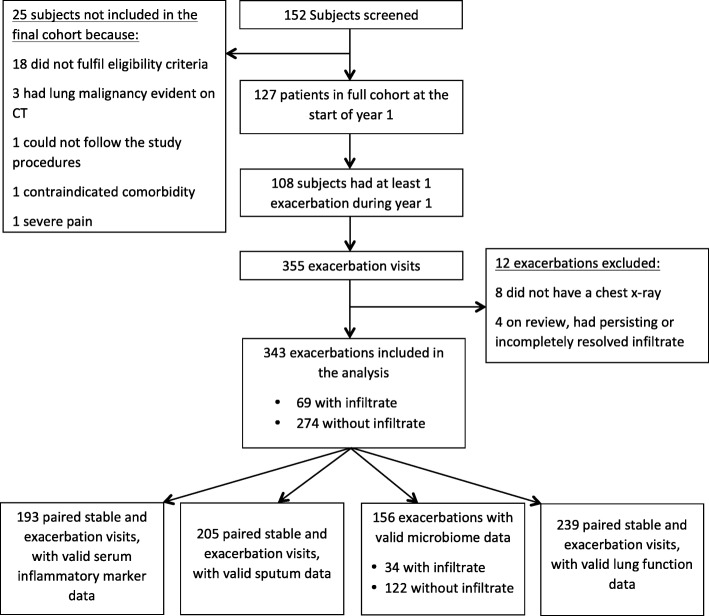
Flow chart of subject enrolment and sub-groupings in the study

One hundred twenty-seven patients were enrolled (Fig. 1). The mean (SD) age was 66.8 ± 8.6 and 68 (53.5%) were male. At study entry 45, 40 and 15% had GOLD stage II, III and IV respectively, with a mean (SD) post-bronchodilator FEV1% predicted of 46.4 ± 15.2 Bronchiectasis, which was assessed at enrolment by high resolution CT, was identified in only a minority of patients (10 of 127; 7.9%) (Table 1).

**Table 1:** Baseline characteristics of the study cohort at the time of recruitment

Characteristic	*n* = 127
Age (years)	66.8 ± 8.6
Male sex, n (%)	68 (53.5)
Body mass index (kg/m^2^)	27.7 (5.5)
Smoking status (n, %)	
Current smoker	54 (43.0)
Ex-smoker	73 (57.0)
Smoking history pack-years^a^	47.0 (26.3)
Post-bronchodilator FEV1 (% predicted)	46.4 ± 15.2
FEV1 (Litres)	1.20 ± 0.47
FVC (Litres)	2.82 ± 0.82
GOLD Stage, n (%)	
II (moderate)	57 (44.9)
III (severe)	51 (40.2)
IV (very severe)	19 (15.0)
Bronchiectasis evident on enrolment HRCT, n (%)	10 (7.9)
Frequency of exacerbation reporting in the preceding 12 months, n (%)	
1 exacerbation	28 (22.0)
2 exacerbations	37 (29.1)
3 or more exacerbations	62 (48.8)
Number of exacerbations in the preceding 12 months	3.1 ± 2.3
ICS use, n (%)	113 (89.0)
LABA use, n (%)	104 (81.9)
LAMA use, n (%)	93 (73.2)
Influenza vaccination in the preceding 12 months, n (%)	114 (89.8)
Pneumococcal vaccination in the preceding 12 months, n (%)	12 (9.4)

Data presented as mean ± standard deviation or n (%) unless otherwise stated

^a^presented as median (IQR)

*FEV1* forced expiratory volume in 1 s, *FVC* forced vital capacity, *GOLD* Global Initiative for Chronic Obstructive Lung Disease, *HRCT* high resolution CT, *ICS* inhaled corticosteroid, *LABA* long acting beta agonist, *LAMA* long acting muscarinic antagonist

Three hundred fifty-five exacerbation visits from 108 patients were studied. The mean annual exacerbation rate was 3.04 (95% CI 2.63–3.50) per patient. 8 exacerbations did not have a CXR and 4 had evidence of persisting infiltrate and were therefore excluded, leaving 343 exacerbations for analysis. Only 19 (5.5%) of these events required hospitalisation. The Kappa agreement for the subset of CXRs analysed independently by two clinical reviewers was 0.968.

Overall, 69 (20.1%) exacerbations had a new pneumonic infiltrate (For other radiological findings, see Additional file 2: Table S1). 61 (88%) infiltrates were considered reticulo-nodular and 8 (12%) lobar in character. 95.7% of exacerbations with pneumonic infiltrate were outpatient managed. 43 were treated with both antibiotics and OCS, 11 with antibiotics alone, 10 with OCS alone and 5 required no systemic treatment (Additional file 2: Table S2). Table 2 shows the clinical characteristics of exacerbations.

**Table 2:** Clinical characteristics of all exacerbations, stratified by the presence or absence of pneumonic infiltrate

	All exacerbations (*n* = 343)			Exacerbations without infiltrate (*n* = 274)			Exacerbations with infiltrate(*n* = 69)			*P*-value*
	n^a^			n^a^			n^a^			
FEV1 (Litres)	311	0.96	(0.62)	255	1.0	(0.62)	56	0.85	(0.68)	0.042
FEV1 (% predicted)	311	40.2	(18.7)	255	41.0	(19.1)	56	37.1	(19.4)	0.038
FEV1/FVC ratio	311	0.39	(0.17)	255	0.39	(0.18)	56	0.35	(0.15)	0.075
CAT score	334	22	(10)	265	22	(10)	69	21	(10)	0.568
EXACT score	318	41	(10)	251	42	(9)	67	40	(11)	0.791
Blood WCC (cellsx10^9^L)	328	8.65	(3.98)	261	8.30	(3.60)	67	9.70	(4.90)	< 0.001
Blood neutrophils (cellsx10^9^L)	328	5.75	(3.50)	261	5.50	(3.10)	67	7.30	(4.50)	< 0.001
Blood eosinophils (cellsx10^9^L)	328	0.20	(0.20)	261	0.20	(0.20)	67	0.20	(0.20)	0.715
% blood eosinophils	328	1.98	(2.43)	261	2.04	(2.43)	67	1.69	(2.28)	0.155
CRP (mg/L)	335	10.0	(23.0)	268	8.0	(16.8)	67	25.0	(61.0)	< 0.001
Fibrinogen (g/L)	314	5.3	(1.8)	250	5.2	(1.5)	64	6.2	(2.0)	< 0.001
Procalcitonin (μg/L)	327	0.0767	(0.0400)	260	0.0751	(0.0400)	67	0.0835	(0.0400)	0.054
Sputum bacterial detection (culture) n, (%)	308	184	(59.7)	245	140	(57.1)	63	44	(69,8)	0.067
Sputum bacterial detection (PCR) n, (%)	295	200	(67.8)	234	153	(65.4)	61	47	(77.0)	0.082
Sputum viral detection (PCR) n, (%)	294	126	(42.9)	234	101	(43.2)	60	25	(41.7)	0.835

Continuous variables presented as median (IQR) and categorical variables as n, (%) unless otherwise stated

*FEV1* Forced expiratory volume in 1 s, *FVC* Forced vital capacity, *CAT* COPD Assessment Test, *EXACT* EXAcerbations of Chronic pulmonary disease Tool, *PCR* Polymerase chain reaction, *WCC* white blood cell count, *CRP* C-reactive protein

^a^indicates the number of subjects with available data/samples

*P-values comparing exacerbations with and without infiltrate using Mann Whitney U test for continuous variables and Chi-square test for categorical variables

Forty-six (36.2%) patients had ≥1 exacerbation with pneumonic infiltrate and 81 (63.8%) did not. In order to identify baseline factors that may predict risk of future exacerbations with infiltrate, these groups were compared. No significant differences in age, sex or smoking status were observed and ICS use was similar. However, greater disease severity (FEV1 (%) 41.7 versus 49.2; *p* = 0.007) and poorer six minute walk distance (256.6 m versus 320.1 m; *p* = 0.002) were associated with having one or more exacerbations with pneumonic infiltrate during the study (Table 3).

**Table 3:** Baseline characteristics of subjects grouped by the presence/absence of at least one exacerbation with pneumonic infiltrate

Characteristic	Subjects with no pneumonic infiltrate identified at any exacerbation (*n* = 81)			Subjects with ≥1 pneumonic infiltrate identified at exacerbation (*n* = 46)			*P*-value
	n^a^			n^a^			
Age (years)	81	66.9	(8.4)	46	66.6	(9.1)	0.843
Male sex, n (%)	81	44	(54.3)	46	24	(52.2)	0.855
Current smokers, n (%)	81	34	(42.0)	46	20	(43.5)	0.508
Body mass index (kg/m^2^)	81	27.8	(5.5)	46	27.5	(5.5)	0.764
FEV1 (Litres)	80	1.26	0.47	46	1.08	(0.99)	0.036
FEV1 (% predicted)	80	49.2	(15.7)	46	41.7	(13.0)	0.007
TLCO (mmol/kPa/min)*	78	4.99	(2.78)	44	3.85	(1.30)	0.005
6MWD (meters)	79	320.1	(110.8)	46	256.6	(100.9)	0.002
CAT score	80	16.3	(7.8)	46	17.5	(7.3)	0.399
Prior history of pneumonia (before study enrolment)	79	22	(27.8)	46	21	(45.7)	0.043
Influenza vaccination during the year prior to enrolment	81	72	(88.9)	46	42	(91.3)	0.786
ICS use	81	69	(85.2)	46	44	(95.7)	0.083
Bacteria detected (culture)	69	34	(49.3)	41	23	(56.1)	0.311
Bacteria detected (PCR)	62	35	(56.5)	40	28	(70.0)	0.169

Continuous variables presented as mean (SD) and categorical variables as n (%) unless otherwise stated. * presented as median (IQR)

*FEV1* forced expiratory volume in 1 s, *TLCO* transfer factor for carbon monoxide, *6MWD* 6 min walk distance, *CAT* COPD Assessment Test, *ICS* inhaled corticosteroid, *PCR* polymerase chain reaction

^a^indicates the number of subjects with available data/samples for each characteristic

*P-values comparing the subject groups using the independent sample t-test or Mann Whitney U test where appropriate for continuous variables and the Chi-square test for categorical variables

### Seasonality of exacerbation groups

**Fig. 2:**
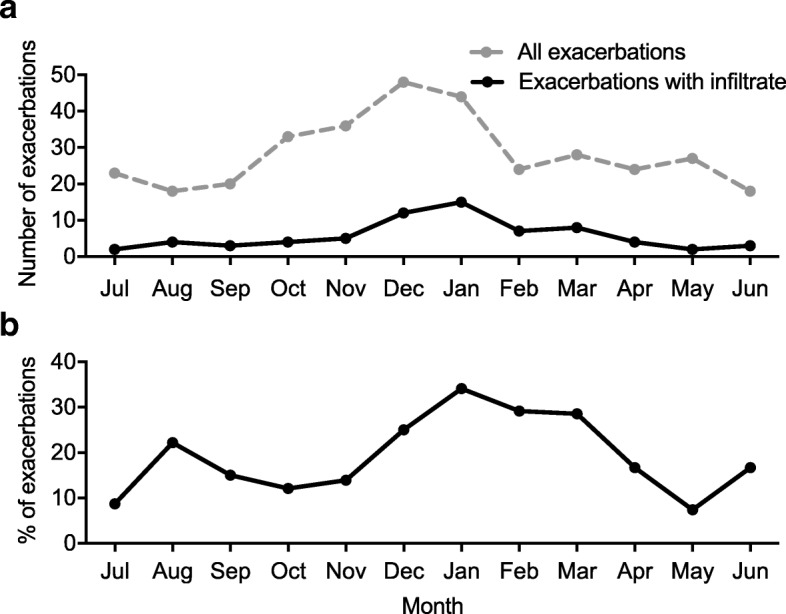
The seasonal distribution of all exacerbations and exacerbations with infiltrate. **a**.) Total number of exacerbations, and number of exacerbations with infiltrate by month; **b**.) Proportion of total exacerbations, with infiltrate by month

Considering all exacerbations (*n* = 343), significant variability was observed across the seasons, with 33.8% occurring in winter, 25.9% in autumn, 23.0% in spring and 17.2% in summer (*p* < 0.001) (Fig. 2a). Exacerbations with infiltrate were highly prevalent in winter, constituting almost a third of all exacerbations during that season (Fig. 2b).

### Pneumonic infiltrates and microbial aetiology

308 (89.8%) exacerbations had available sputum for bacterial culture, 295 (86.0%) for bacterial PCR and 294 (85.7%) for viral PCR. Overall, 69.8% of exacerbations with pneumonic infiltrate and 57.1% of exacerbations without had bacteria detected by culture (*p* = 0.067). For PCR-detected bacteria, corresponding proportions were 77.0 and 65.4% respectively for exacerbations with and without pneumonic infiltrate (*p* = 0.082). *Haemophilus influenzae* (HI) was the most frequently identified bacteria by both culture (52.4% of exacerbations with, versus 38.0% without infiltrate, *p* = 0.038) and PCR (62.3% of exacerbations with, versus 52.4% without infiltrate, *p* = 0.165). A separate analysis, including only sputum considered high quality (fewer than 30% squamous cells) yielded similar results (Additional file 2: Table S3). *Streptococcus pneumoniae* (SP) was detected by culture in 11.1% (*n* = 7) of exacerbations with, and 16.3% (*n* = 40) without pneumonic infiltrate (*p* = 0.305) (Additional file 2: Table S4 and Figure S1). PCR detected only 19 (8.2%) cases of SP at exacerbations without infiltrate, accounted for by the identification of the closely related species, *Streptococcus pseudopneumoniae* by PCR, which was not differentiated by culture [6]. Of those with lobar infiltrate (*n* = 8), 5 (62.5%) were associated with bacterial detection, 4 of which were HI, whilst SP was not detected.

Viruses were identified in similar proportions between exacerbation groups (*n* = 25 (41.7%) with, versus *n* = 101 (43.2%) without infiltrate; *p* = 0.835), with rhinovirus predominating. There were no differences in bacterial-viral co-infection rates by either bacterial culture-viral PCR (*n* = 18 (30.0%) with, versus *n* = 58 (24.8%) without infiltrate; *p* = 0.411) or bacterial PCR-viral PCR (*n* = 21 (35.0%) with, versus *n* = 68 (29.1%) without; *p* = 0.372).

Using a subset of 205 exacerbations with paired preceding stable visits and available sputum, (*n* = 38 (18.5%) with and *n* = 167 (81.5%) without infiltrate), acquisition of bacteria not seen at the previous stable visit occurred in 37.6%. There were no differences in the rate of new bacterial acquisition between exacerbations with and without pneumonic infiltrate (*n* = 15 (39.5%) versus *n* = 62 (37.1%) respectively; *p* = 0.787).

### Pneumonic infiltrates and the microbiome

**Fig. 3:**
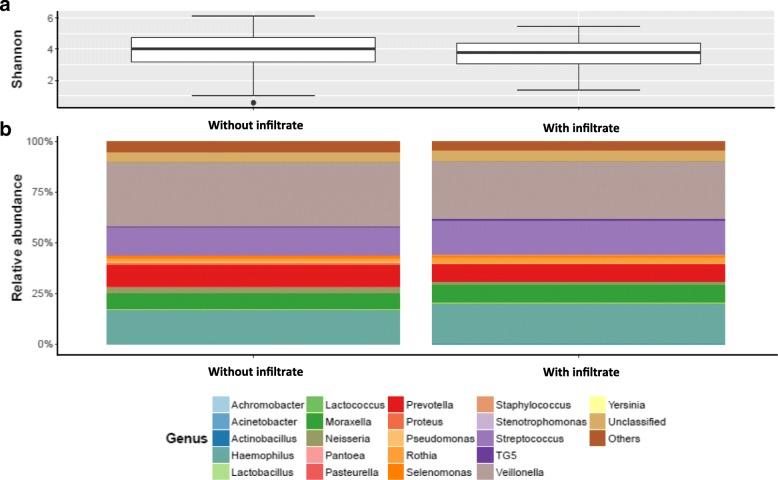
The lung microbiome of exacerbations, stratified by the presence or absence of pneumonic infiltrate. **a**.) The Shannon diversity index did not show any significant difference between radiological groups (*p* = 0.34). **b**.) The genus-level abundances showed no significant differences between groups (*p* = 0.54)

Firmicutes, Proteobacteria and Bacteroidetes were the most abundant phyla and Veillonella, Haemophilus, Streptococcus and Prevotella the most abundant genera identified at exacerbation. Stratifying by presence or absence of pneumonic infiltrate did not identify any significant differences in the main phyla (*p* = 0.350), genera (*p* = 0.540) nor in Shannon diversity between exacerbation groups. (Fig. 3 and Additional file 2: Figure S2).

### Pneumonic infiltrates and inflammatory responses

When considering all exacerbations, those with pneumonic infiltrates had significantly greater systemic inflammation (*p* < 0.001 for CRP, fibrinogen and neutrophil count), than those without (Table 2). In exacerbations with available high quality sputum (*n* = 209), pneumonic infiltrate was associated with an increased sputum neutrophil percentage (89.6 versus 79.1, *p* = 0.007). CRP had the greatest area under the receiver operator curve (0.692) for distinguishing exacerbations with infiltrate (Additional file 2: Figure S3).

**Fig. 4:**
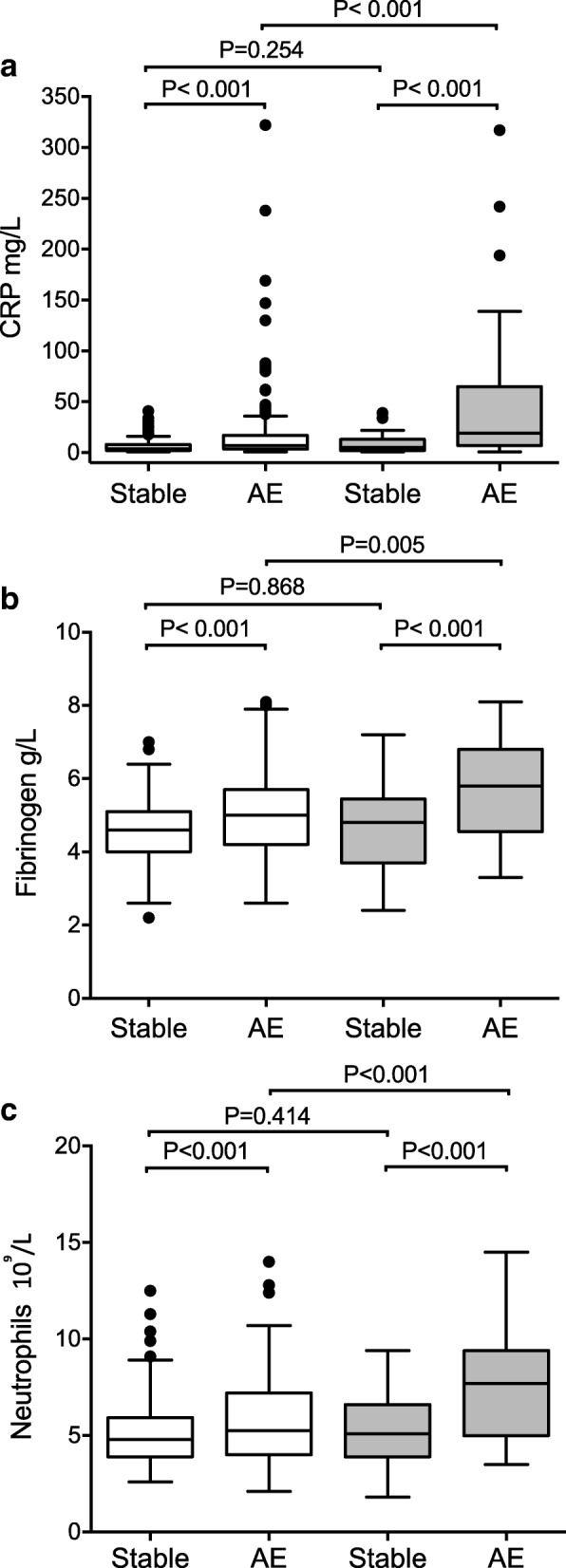
Blood inflammatory marker levels at exacerbations, stratified by the presence or absence of pneumonic infiltrate. Box and whisker plots showing **a**.) Blood C-reactive protein (CRP) levels; **b**.) Blood fibrinogen levels and **c**.) Blood neutrophil counts, in paired stable and acute exacerbation (AE) samples. White bars represent paired stable, non-infiltrate associated AE visits and grey bars represent paired stable, infiltrate-associated AE visits. Data are presented as median (interquartile range)

Inflammation can vary at baseline, therefore a paired analysis comparing pre-exacerbation (stable visit) with exacerbation samples was performed for 193 exacerbations. The median (IQR) time between stable and exacerbation samples was 42 (39) days for those with pneumonic infiltrate and 38 (46) days for those without (*p* = 0.328). All markers analysed, increased at exacerbation. However, exacerbations with pneumonic infiltrate had a significantly higher median CRP (19.0 versus 7.0 mg/L; *p* < 0.001), fibrinogen (5.8 versus 5.0 g/L; *p* < 0.005), blood neutrophil count (7.7 versus 5.3 10^9^L; *p* < 0.001) (Fig. 4 and Additional file 2: Table S5) and greater median changes from stable-state than those without (Additional file 2: Table S6). For PCT, although greater increases were observed from stable-state in those exacerbations with infiltrate compared to those without, PCT was otherwise not a good discriminatory marker. A separate paired analysis (Additional file 2: Table S7), using only the first infiltrate-associated exacerbation where available, or the first non-infiltrative exacerbation where not (subjects were therefore represented only once), yielded similar results. Infiltrate character did not appear to influence inflammatory responses.

### Lung function changes at exacerbation

Paired lung function was available for 239 exacerbations. To highlight clinical severity, infiltrate-associated exacerbations were associated with greater falls in FEV1 than those without (− 125 ml versus -40 ml, *p* = 0.010). A similar trend was also seen for FVC, although this did not reach significance (Additional file 2: Table S8).

### Predicting pneumonic infiltrate at exacerbation

Factors significantly associated with increased odds of having pneumonic infiltrate at exacerbation were winter season (OR 3.056, 95%CI 1.139–8.200, *p* = 0.027) and greater CRP levels (categorised by tertiles) at exacerbation (OR 5.723, 95%CI 2.273–14.407, *p* < 0.001 for a CRP > 18 versus < 6 mg/L). Cold and/or sore throat symptoms were associated with reduced odds of having pneumonic infiltrate (OR 0.435, 95%CI 0.223–0.847, *p* = 0.014) (Table 4).

**Table 4:** Risk factors associated with the odds of pneumonic infiltrate at exacerbation

Characteristic	OR	(95% CI)	*P*-value
Age (per year)	0.996	(0.952–1.042)	0.869
Male sex	1.036	(0.501–2.143)	0.923
BMI (per 1 kg/m^2^)	0.995	(0.938–1.056)	0.938
Current smoker	1.524	(0.700–3.319)	0.288
ICS use	3.552	(0.322–39.159	0.301
Maintenance bronchodilator use^a^	1.253	(0.182–8.641)	0.819
FEV1% predicted			
≥ 50%	Reference		
30- < 50%	1.044	(0.496–2.198)	0.909
< 30%	1.987	(0.763–5.174)	0.159
Season			
Spring (Mar – May)	1.435	(0.498–4.134)	0.504
Summer (Jun – Aug)	Reference		
Autumn (Sep – Nov)	1.198	(0.407–3.523)	0.743
Winter (Dec – Feb)	3.056	(1.139–8.200)	0.027
Sputum purulence	1.112	(0.572–2.162)	0.755
Fever	2.583	(0.979–6.811)	0.055
Cold and/or sore throat	0.435	(0.223–0.847)	0.014
Blood eosinophils < 2% at exacerbation	0.871	(0.451–1.681)	0.680
CRP < 6 mg/L	Reference		
CRP 6–18 mg/L	2.886	(1.123–7.419)	0.028
CRP > 18 mg/L	5.723	(2.273–14.407)	< 0.001

*BMI* body mass index, *FEV1* forced expiratory volume in 1 s, *CRP* C-reactive protein

Clustered multivariate logistic regression analysis, including the patient as a random effect, for 313 exacerbations (63 with infiltrate, 250 without) with complete data for all the variables included in the model. Other independent variables included in the regression model were influenza and pneumococcal vaccine in the year prior to enrolment

^a^refers to the use of either a long acting beta agonist or long acting muscarinic antagonist, alone or in combination with other inhaled medication

CRP ranges based on categorisation by tertiles

## Discussion

In this prospective study of outpatients with moderate to very severe COPD, we show that exacerbations with new pneumonic infiltrates were associated with heightened levels of systemic inflammation, greater lung function deterioration and similar microbiological signatures, compared to exacerbations without. Importantly, more than 94% of exacerbations were managed as an outpatient, therefore providing a unique insight into the incidence and significance of pneumonic infiltrate in this clinical setting.

Recent interest has focused on understanding the heterogeneity of exacerbations [8]. Although the use of CXR has been reported in hospitalised COPD patients [13, 21, 22], few prospective studies report on its use in such a well-characterised outpatient COPD cohort, and none addressing the microbiological and inflammatory complexities of exacerbations to the extent of the AERIS study. The only other large COPD study collecting radiological data at exacerbation, did not report on inflammatory or microbiological indices [23]. Although having a similar patient demographic, the differing primary objectives and aims make direct comparisons to our study difficult.

Pneumonic infiltrates were a feature in approximately 20% of exacerbations. Furthermore, half of all infiltrate-associated exacerbations occurred during the winter, and proportionally were a feature in around a third of all exacerbations occurring during that period, therefore contributing considerably to the exacerbation-burden during this time of year. In multivariate analysis seasonality remained an important determinant, with greater odds of having an exacerbation with infiltrate in winter, compared to summer. The seasonal nature of exacerbations and of pneumonia in COPD has previously been described [6, 11, 24, 25]. Population behaviours, changes in temperature and humidity, as well as cyclical patterns of respiratory pathogens have all been proposed as key determinants [26]. Understanding whether seasonality could direct exacerbation treatment approaches remains to be seen, but characterising exacerbations using methods such as CXR could guide this approach.

Exacerbations are typically inflammatory events [5]. In this study, exacerbations with pneumonic infiltrate were associated with greater airway neutrophillic inflammation and showed more intense systemic inflammatory responses than exacerbations without. Additionally, significant decreases in FEV1 at exacerbation onset were seen in those with pneumonic infiltrate. This observed decline, provides a measure of exacerbation severity and may be directly associated with the greater inflammatory responses identified. Neutrophilic inflammation has previously been shown to correlate with greater decreases in lung function during exacerbation [27, 28], but these studies did not utilise radiological stratification.

The intensity of systemic inflammation at exacerbation has previously been linked to the presence of bacteria [4], bacterial strain changes [29] and bacterial-viral co-infection [28]. In agreement with prior studies [30, 31], we have previously reported a substantial number of exacerbations associated with viral detection within this cohort [6]. However, overall the proportion of viral-associated exacerbations with and without pneumonic infiltrate was broadly similar and viral symptoms (cold and/or sore throat) occurred less frequently in exacerbations with pneumonic infiltrate.

Bacteria, particularly HI, have long been regarded as an important cause of exacerbations [32]. Conversely, studies of hospitalised pneumonia in COPD patients consistently report SP as the most frequently implicated bacteria [13, 22], similarly for hospitalised pneumonia in patients without COPD [15]. Like previous studies, our data re-confirms HI as the most frequently detected bacteria at exacerbation, but additionally suggests a potential association between HI and pneumonic infiltrate. By culture, HI was implicated in 52% of exacerbations with infiltrate compared to 38% of those without. However, as HI commonly causes chronic infection in COPD even at stable-state, establishing a causal link between HI detection at exacerbation and risk of pneumonic infiltrate is challenging. In this regard, although new bacterial acquisition did not appear to be associated with pneumonic infiltrate, bacterial strain changes and increasing load of existing pathogens could be key mechanisms, having previously been implicated in exacerbation development and more profound inflammatory responses [28, 29].

This study adds to the evolving exploration of exacerbation heterogeneity, using radiological stratification in an outpatient COPD population. Current practise varies on whether an acute respiratory illness, complicated by pneumonic infiltrate in COPD patients is included under the diagnosis of exacerbation or considered as pneumonia. There is some evidence to suggest that exacerbations and pneumonia in COPD differ in their aetiology and inflammatory profile and should perhaps be considered distinct, despite being clinically hard to distinguish [13, 33]. Our findings suggest that the infective aetiology of exacerbations is similar to that of acute events classified as pneumonia in an outpatient clinical setting. The results suggest common mechanisms between pneumonia and exacerbations in patients with COPD and will help to inform exacerbation management.

We recognise that this study has limitations. The use of CXR remains the radiological standard for diagnosing pneumonia, and yet false-positive and false-negative results can occur [34]. CT scanning, may improve diagnostic accuracy, but is currently more expensive, less readily available and associated with greater radiation dose than plain CXR, making its utilisation currently unfeasible in a study of frequent exacerbators.

The mean exacerbation rate in AERIS reflects the selected cohort of patients with a history of frequent exacerbations. We accept that our findings may not be generalizable to more heterogeneous groups, or those that infrequently exacerbate. Nevertheless, like other studies, the exacerbation rate for the cohort was similar to that in the year prior to enrolment, suggesting a fairly constant phenotype rather than an overestimation of exacerbations [35, 36].

There are well-established links between ICS use and increased pneumonia risk in COPD [10, 37]. In our cohort, the majority of patients were already prescribed ICS at enrolment, limiting our ability to assess the impact of ICS use on the risk of developing pneumonic infiltrates. We also acknowledge that differing methodological approaches for determining aetiology, may limit direct comparisons with other studies. However, like a recent report of hospitalised pneumonia, not limited to COPD patients [38], we utilised sputum for microbiological analysis and similarly identified HI as the most frequently detected bacteria. This contrasts with many other studies of COPD patients hospitalised with pneumonia, where an aetiological diagnosis was predominantly made from blood culture, urinary antigen testing or serological analysis and was typically found to be SP [13, 21]. However, due to the early identification and treatment of exacerbations, we cannot exclude the possibility that bacteria such as SP may have a role in the later stages of pneumonic exacerbations. Additionally, the use of electronic diary cards and consequently the early capture of exacerbations, may underestimate the frequency of complicating pneumonic infiltrates, as radiological changes in the context of acute lower respiratory tract illness can lag behind clinical symptoms [39].

## Conclusions

This is the first prospective study to radiologically stratify exacerbations and demonstrate the incidence, seasonality and character of acute events characterised as pneumonia in an outpatient COPD cohort. That pneumonia is common in this clinical context, associated with heightened levels of systemic inflammation, greater lung function deterioration and a similar infective aetiology to exacerbations, is highly relevant clinically. These results suggest that exacerbations and pneumonia in COPD share common infectious triggers and represent a continuum of severity rather than distinct aetiological events. This knowledge will help to inform exacerbation management, particularly important in an era of emerging antimicrobial resistance and antibiotic governance. Based on these findings, interventional studies, testing the utility of radiological and inflammatory stratification of exacerbations to assess optimal response to standard treatments are required.

## Additional files

Additional file 1: AERIS study subject inclusion and exclusion criteria and supplementary methods. (DOCX 58 kb)

Additional file 2: **Table S1.** Radiological findings at exacerbation. **Table S2.** Exacerbation treatment stratified by the presence or absence of radiographic pneumonic infiltrate. **Table S3.** Bacterial identification by culture and PCR in all exacerbation sputum samples and those exacerbation sputum samples with fewer than 30% squamous cells (considered high quality). **Table S4.** Bacterial and viral identification at exacerbation by culture (bacteria) and PCR (bacteria/viral). **Table S5.** Levels of inflammatory markers at paired stable and exacerbation visits. **Table S6.** Changes in levels of serum inflammatory markers between stable (pre-exacerbation) and exacerbation samples. **Table S7.** Levels of serum inflammatory markers at paired stable and exacerbation visits. The occurrence of the first infiltrate-associated exacerbation where available was prioritised, or first non-infiltrative exacerbation if not (subjects are therefore only represented once). **Table S8.** Lung function changes between nearest stable-state and exacerbation visits, stratified by the presence/absence of pneumonic infiltrate. **Figure S1.** The proportion of bacterial positive sputum samples at exacerbation by both culture and PCR. **Figure S2.** The lung microbiome (phylum) of exacerbations stratified by the presence or absence of pneumonic infiltrate. **Figure S3.** Area under the receiver operator curve analysis for CRP, fibrinogen and neutrophil count. (DOCX 232 kb)

## Abbreviations

AERIS: Acute Exacerbations and Respiratory Infections in COPD study
CI: Confidence interval
COPD: Chronic obstructive pulmonary disease
CRP: C-reactive protein
CT: Computed tomography
CXR: Chest radiograph
FEV1: Forced expiratory volume in 1 s
FVC: Forced vital capacity
GOLD: Global initiative for chronic obstructive lung disease
HI: *Haemophilus influenzae*
ICS: Inhaled corticosteroid
IQR: Interquartile range
OCS: Oral corticosteroids
OR: Odds ratio
OTU: Operational taxanomic units
PCR: Polymerase chain reaction
PCT: Procalcitonin
SD: Standard deviation
SP: *Streptococcus pneumoniae*

## References

[1] Miravitlles M, Ferrer M, Pont A, Zalacain R, Alvarez-Sala JL, Masa F, Verea H, Murio C, Ros F, Vidal R (2004). Effect of exacerbations on quality of life in patients with chronic obstructive pulmonary disease: a 2 year follow up study. Thorax.

[2] Donaldson GC, Seemungal TA, Bhowmik A, Wedzicha JA (2002). Relationship between exacerbation frequency and lung function decline in chronic obstructive pulmonary disease. Thorax.

[3] Anzueto A (2010). Impact of exacerbations on COPD. Eur Respir Rev.

[4] Hurst JR, Perera WR, Wilkinson TM, Donaldson GC, Wedzicha JA (2006). Systemic and upper and lower airway inflammation at exacerbation of chronic obstructive pulmonary disease. Am J Respir Crit Care Med.

[5] Bathoorn E, Kerstjens H, Postma D, Timens W, MacNee W (2008). Airways inflammation and treatment during acute exacerbations of COPD. Int J Chron Obstruct Pulmon Dis.

[6] Wilkinson TMA, Aris E, Bourne S, Clarke SC, Peeters M, Pascal TG, Schoonbroodt S, Tuck AC, Kim V, Ostridge K, et al. A prospective, observational cohort study of the seasonal dynamics of airway pathogens in the aetiology of exacerbations in COPD. Thorax. 2017; 10.1136/thoraxjnl-2016-209023 PMC5738531 28432209

[7] Miravitlles M, Murio C, Guerrero T (2001). Factors associated with relapse after ambulatory treatment of acute exacerbations of chronic bronchitis. DAFNE Study Group Eur Respir J.

[8] Bafadhel M, McKenna S, Terry S, Mistry V, Reid C, Haldar P, McCormick M, Haldar K, Kebadze T, Duvoix A (2011). Acute exacerbations of chronic obstructive pulmonary disease: identification of biologic clusters and their biomarkers. Am J Respir Crit Care Med.

[9] Torres A, Peetermans WE, Viegi G, Blasi F (2013). Risk factors for community-acquired pneumonia in adults in Europe: a literature review. Thorax.

[10] Crim C, Calverley PM, Anderson JA, Celli B, Ferguson GT, Jenkins C, Jones PW, Willits LR, Yates JC, Vestbo J (2009). Pneumonia risk in COPD patients receiving inhaled corticosteroids alone or in combination: TORCH study results. Eur Respir J.

[11] Williams NP, Coombs NA, Johnson MJ, Josephs LK, Rigge LA, Staples KJ, Thomas M, Wilkinson TM (2017). Seasonality, risk factors and burden of community-acquired pneumonia in COPD patients: a population database study using linked health care records. Int J Chron Obstruct Pulmon Dis.

[12] Royal College of Physicians (2015). National Chronic Obstructive Pulmonary Disease Audit Programme: clinical audit of COPD exacerbations admitted to acute units in England and Wales 2014. Royal College of Physicians.

[13] Huerta A, Crisafulli E, Menendez R, Martinez R, Soler N, Guerrero M, Montull B, Torres A (2013). Pneumonic and nonpneumonic exacerbations of COPD: inflammatory response and clinical characteristics. Chest.

[14] Andreassen SL, Liaaen ED, Stenfors N, Henriksen AH (2014). Impact of pneumonia on hospitalizations due to acute exacerbations of COPD. Clin Respir J.

[15] Crisafulli E, Menendez R, Huerta A, Martinez R, Montull B, Clini E, Torres A (2013). Systemic inflammatory pattern of patients with community-acquired pneumonia with and without COPD. Chest.

[16] Donaldson GC, Goldring JJ, Wedzicha JA (2012). Influence of season on exacerbation characteristics in patients with COPD. Chest.

[17] Bourne S, Cohet C, Kim V, Barton A, Tuck A, Aris E, Mesia-Vela S, Devaster JM, Ballou WR, Clarke SC, Wilkinson T (2014). Acute exacerbation and respiratory InfectionS in COPD (AERIS): protocol for a prospective, observational cohort study. BMJ Open.

[18] Kim VL, Coombs NA, Staples KJ, Ostridge KK, Williams NP, Wootton SA, Devaster JM, Aris E, Clarke SC, Tuck AC, et al. Impact and associations of eosinophilic inflammation in COPD: analysis of the AERIS cohort. Eur Respir J. 2017;50 10.1183/13993003.00853-2017 29025891

[19] Ostridge K, Williams NP, Kim V, Harden S, Bourne S, Clarke SC, Aris E, Mesia-Vela S, Devaster JM, Tuck A (2018). Relationship of CT-quantified emphysema, small airways disease and bronchial wall dimensions with physiological, inflammatory and infective measures in COPD. Respir Res.

[20] Mayhew D, Devos N, Lambert C, Brown JR, Clarke SC, Kim VL, Magid-Slav M, Miller BE, Ostridge KK, Patel R, et al. Longitudinal profiling of the lung microbiome in the AERIS study demonstrates repeatability of bacterial and eosinophilic COPD exacerbations. Thorax. 2018; 10.1136/thoraxjnl-2017-210408 PMC5909767 29386298

[21] Molinos L, Clemente MG, Miranda B, Alvarez C, del Busto B, Cocina BR, Alvarez F, Gorostidi J, Orejas C (2009). Community-acquired pneumonia in patients with and without chronic obstructive pulmonary disease. J Infect.

[22] Liapikou A, Polverino E, Ewig S, Cilloniz C, Marcos MA, Mensa J, Bello S, Martin-Loeches I, Menendez R, Torres A (2012). Severity and outcomes of hospitalised community-acquired pneumonia in COPD patients. Eur Respir J.

[23] Crim C, Dransfield MT, Bourbeau J, Jones PW, Hanania NA, Mahler DA, Vestbo J, Wachtel A, Martinez FJ, Barnhart F (2015). Pneumonia risk with inhaled fluticasone furoate and vilanterol compared with vilanterol alone in patients with COPD. Ann Am Thorac Soc.

[24] Jenkins CR, Celli B, Anderson JA, Ferguson GT, Jones PW, Vestbo J, Yates JC, Calverley PM (2012). Seasonality and determinants of moderate and severe COPD exacerbations in the TORCH study. Eur Respir J.

[25] Rabe KF, Fabbri LM, Vogelmeier C, Kogler H, Schmidt H, Beeh KM, Glaab T (2013). Seasonal distribution of COPD exacerbations in the prevention of exacerbations with tiotropium in COPD trial. Chest.

[26] Donaldson GC, Wedzicha JA (2014). The causes and consequences of seasonal variation in COPD exacerbations. Int J Chron Obstruct Pulmon Dis.

[27] Papi A, Bellettato CM, Braccioni F, Romagnoli M, Casolari P, Caramori G, Fabbri LM, Johnston SL (2006). Infections and airway inflammation in chronic obstructive pulmonary disease severe exacerbations. Am J Respir Crit Care Med.

[28] Wilkinson TM, Hurst JR, Perera WR, Wilks M, Donaldson GC, Wedzicha JA (2006). Effect of interactions between lower airway bacterial and rhinoviral infection in exacerbations of COPD. Chest.

[29] Sethi S, Wrona C, Eschberger K, Lobbins P, Cai X, Murphy TF (2008). Inflammatory profile of new bacterial strain exacerbations of chronic obstructive pulmonary disease. Am J Respir Crit Care Med.

[30] Seemungal T, Harper-Owen R, Bhowmik A, Moric I, Sanderson G, Message S, Maccallum P, Meade TW, Jeffries DJ, Johnston SL, Wedzicha JA (2001). Respiratory viruses, symptoms, and inflammatory markers in acute exacerbations and stable chronic obstructive pulmonary disease. Am J Respir Crit Care Med.

[31] McManus TE, Marley AM, Baxter N, Christie SN, O'Neill HJ, Elborn JS, Coyle PV, Kidney JC (2008). Respiratory viral infection in exacerbations of COPD. Respir Med.

[32] Sethi S (2004). Bacteria in exacerbations of chronic obstructive pulmonary disease: phenomenon or epiphenomenon?. Proc Am Thorac Soc.

[33] Gutierrez P, Closa D, Piner R, Bulbena O, Menendez R, Torres A (2010). Macrophage activation in exacerbated COPD with and without community-acquired pneumonia. Eur Respir J.

[34] Claessens YE, Debray MP, Tubach F, Brun AL, Rammaert B, Hausfater P, Naccache JM, Ray P, Choquet C, Carette MF (2015). Early chest computed tomography scan to assist diagnosis and guide treatment decision for suspected community-acquired pneumonia. Am J Respir Crit Care Med.

[35] Hurst JR, Vestbo J, Anzueto A, Locantore N, Mullerova H, Tal-Singer R, Miller B, Lomas DA, Agusti A, Macnee W (2010). Susceptibility to exacerbation in chronic obstructive pulmonary disease. N Engl J Med.

[36] Mullerova H, Shukla A, Hawkins A, Quint J (2014). Risk factors for acute exacerbations of COPD in a primary care population: a retrospective observational cohort study. BMJ Open.

[37] Calverley PM, Stockley RA, Seemungal TA, Hagan G, Willits LR, Riley JH, Wedzicha JA (2011). Reported pneumonia in patients with COPD: findings from the INSPIRE study. Chest.

[38] Gadsby NJ, Russell CD, McHugh MP, Mark H, Conway Morris A, Laurenson IF, Hill AT, Templeton KE (2016). Comprehensive molecular testing for respiratory pathogens in community-acquired pneumonia. Clin Infect Dis.

[39] Hagaman JT, Rouan GW, Shipley RT, Panos RJ (2009). Admission chest radiograph lacks sensitivity in the diagnosis of community-acquired pneumonia. Am J Med Sci.

