# Supplementary material for: Impact of radiologically stratified exacerbations: insights into pneumonia aetiology in COPD
Source: Respir Res. 2018 Jul 28;19:143. doi: 10.1186/s12931-018-0842-8 (PMC6064093; doi:10.1186/s12931-018-0842-8)
Supplement: Supplementary file 1 — AERIS study subject inclusion and exclusion criteria and supplementary methods. (DOCX 58 kb) [file 12931_2018_842_MOESM1_ESM.docx]

**Online supplement for:**

**Impact of Radiologically Stratified Exacerbations: Insights into Pneumonia Aetiology in COPD**

Nicholas P Williams,^1,2^ Kristoffer Ostridge,^1,2^ Jeanne-Marie Devaster,^3^ Viktoriya Kim,^1,2^ Ngaire A Coombs,^4^ Simon Bourne,^1,^* Stuart C Clarke,^1,5^ Stephen Harden,^6^ Ausami Abbas,^6^ Emmanuel Aris,^3^ Christophe Lambert,^3^ Andrew Tuck,^7^ Anthony Williams,^5^ Stephen Wootton,^8^ Karl J Staples,^1,5^ Tom MA Wilkinson,^1,2,5^ on behalf of the AERIS Study Group^‡^

**Inclusion and exclusion criteria**

All subjects must satisfy all the following criteria at study entry:

- Subjects who the investigator believes can and will comply with the requirements of the protocol.
- Written informed consent obtained from the subject.
- Male or female subjects between, and including, 40 and 85 years of age, at the time of consent.
- Subjects with confirmed diagnosis of COPD (based on post-bronchodilator spirometry) with forced expiratory volume of air expired in 1 second (FEV1) of ≤80% of predicted normal and FEV1/forced expiratory vital capacity <0·7.
- Subjects have moderate, severe, or very severe COPD, according to Global Initiative for Chronic Obstructive Lung Disease (GOLD) staging.
- Subjects have a current or prior history of 10 pack-years of cigarette smoking. Former smokers are defined as those who have stopped smoking for at least 6 months. Number of pack years = (number of cigarettes per day/20) x number of years smoked.
- Subjects present a documented history of ≥1 exacerbation requiring antibiotics and/or oral corticosteroids or hospitalization in the previous 12 months.
- Subjects with recent COPD exacerbations, in stable condition, and having stopped antibiotics, can be enrolled one month post exacerbation.

The following criteria should be checked at the time of study entry. If any exclusion criterion applies, the subject must not be included in the study:

- Subject has a confirmed diagnosis of asthma (as only cause of obstructive respiratory disorder), cystic fibrosis, pneumonia risk factors or other respiratory disorders (e.g. tuberculosis, lung cancer).
- Subjects having undergone lung surgery.
- Subject has a α-1 antitrypsin deficiency as underlying cause of COPD.
- Subject who experienced a moderate or severe COPD exacerbation not resolved at least 1 month prior to enrolment visit and at least 30 days following the last dose of oral corticosteroids (subjects can be enrolled when their AECOPD or pneumonia has resolved).
- Subject using any antibacterial, antiviral, or respiratory investigational drug or vaccine up to 30 days prior to the enrolment visit.
- Subject has other conditions that the principal investigator judges may interfere with the study findings, such as:
  - Subject at risk of non-compliance or unable to comply with the study procedures.
  - Evidence of alcohol or drug abuse.
- Women who are pregnant or lactating or are planning on becoming pregnant during the study.

**Supplementary Methods**

**Exacerbations**

Exacerbations were defined as worsening of at least two major symptoms (dyspnoea, sputum volume, and sputum purulence) or worsening of at least one major symptom and one minor symptom (wheeze, sore throat, cold symptoms, cough, and fever without other cause). An exacerbation was considered mild severity if no antibiotic and/or oral corticosteroid (OCS) therapy was required, moderate if treatment with antibiotics and/or OCS or antibiotics was required, and severe if the patient required hospitalisation.

**Sputum collection and processing**

Sputum samples were collected at study entry (enrolment), monthly, and at exacerbation. Within two hours of expectoration, sputum plugs were separated from saliva using sterile forceps. Samples were kept at room temperature and sent to the Public Health England laboratory for culture-based microbiology.

Potential bacterial respiratory pathogens, including *Haemophilus influenzae*, *Moraxella catarrhalis*, *Streptococcus pneumoniae*, *Pseudomonas aeruginosa*, and *Staphylococcus aureus* were identified using conventional culture techniques and by polymerase chain reaction (PCR).

Sputum samples were also analysed for the detection of respiratory viruses by PCR. A qualitative nucleic acid multiplex test was used (xTAG® Respiratory Viral Panel Fast v2; Luminex, Austin, TX, USA), which allows for the simultaneous detection and identification of multiple respiratory virus nucleic acids in respiratory specimens, including: influenza A, including subtypes of influenza A (H1 and H3), and distinguishes between 2009 H1N1 and other H1N1 (seasonal) strains, influenza B, respiratory syncytial virus, human metapneumovirus, parainfluenza virus 1–4, coronavirus (OC43, 229E, NL63, HKU1), rhinovirus/enterovirus, adenovirus, and bocavirus.

**Microbiome analysis**

591 samples were selected for microbiome analysis, 163 of them were taken during visits of patient following AECOPD.

Raw FASTQ sequence files where analyzed separately until the final merge for species assignment. Firstly reads where filtered for PhiX. All reads mapping to *Enterobacteria phage phiX174 senso lato* reference genome (GenBank: NC_001422.1) using bowtie2 v2.1.0 where removed from the analysis.

Remaining reads where merged using pear v0.9.5-64 discarding all reads containing ambiguous bases (option “-u 0”). A paired-end read was discarded if one of the following conditions was met: overlap below 10bp, assembly length<50bp or p-value of alignment > 0.01. Sequences where then analyzed with the QIIME pipeline version 1.8.0. Sequences were assigned to samples on the basis of their MID tag, allowing for no base error. Merged reads shorter than 75 bases were discarded. Demultiplexed reads from the four sequencing runs were merged in a single file. To remove sequencing errors, chimeric reads were identified and removed using the command identify_chimeric_seqs.py from the QIIME pipeline. USEARCH v6.1.544 was used for the alignment and GreenGene v13.8 clustered at 97% level was used as a reference database.

OTUs were subsequently clustered from chimera cleaned reads at a 97%-identity threshold using UCLUST v6.1.544 with default settings and GreenGene v13.8 to assign taxonomy. Abundance matrices of phylogenetic abundance per sample were calculated at different taxonomic levels from OTU and respective taxonomic classifications features.

Samples were rarefied at 29658 reads, and the alpha diversity indexes (Chaos, Simpson reciprocal, Shannon and richness) or weighted and unweighted UniFrac distances were computed. For alpha diversity, 100 rarefactions were simulated and alpha diversity indices computed. For each sample, the reported alpha diversity values were obtained by computed the average value of the 100 simulations. To ensure normality of the distribution, a square root transformation has been performed on Chaos index and a log transform has been performed on Simpson reciprocal.

Data from 5 samples were discarded as they didn’t reach the threshold of 1000 reads equivalent to blank (water only) (2 exacerbation visits and 3 stable visits) and 3 additional samples were not considered for alpha diversity index and Unifrac distance computation as they didn’t reach the threshold of 29658 reads used for rarefaction.

The analysis reported 543 unique taxa for the 586 samples considered for the analysis, with an average of 59.6 taxa per samples. The five most abundant genera represent 82.56% of all reads assigned: *Veillonella* (34.56%), *Haemophilus* (17.88%), *Streptococcus* (16.35%), *Prevotella* (9.69%) and *Moraxella* (4.08%).

Pneumonia was assessed by the presence of pulmonary infiltrate on CXR infiltrate in 160 out of the 163 visits of patient following AECOPD. Two pneumonic events were excluded from the analysis as they were considered non-resolved from a previous pneumonia, and two samples at exacerbation visits had less than 1000 reads. Therefore 156 samples remained for the analysis, 122 with no pneumonic event and 34 with pneumonic events.

Microbiomes of pneumonia and non-pneumonia samples were compared using different measures: phylum and genus relative abundances, Shannon index and weighted and unweighted UniFrac distances.

**Statistical analysis**

**Sample size calculation**

The sample size for the overall AERIS study was based on the primary study endpoint of incidence of all-cause exacerbations. It was assumed that on average, each participant would be observed for a period of 18 months and that two exacerbation episodes can be expected per participant per year. If 120 participants are followed, the number of total person-years would be approximately 180 and during this time approximately 360 exacerbation events would occur. If the distribution of events per participant follows a Poisson distribution with no over-dispersion, an over-dispersion factor of 1.5, or an over-dispersion of 2, the approximate values of the lower and upper bounds of the 95% CI around the point estimate of two events per participant per year would be 1.8–2.2, 1.7–2.3 and 1.7–2.3, respectively. So a sample size of 120 participants should ensure a sufficient estimation of the incidence rate of all-cause exacerbations (1).

**Microbiome**

The statistical analysis for the microbiome data was performed using R version 3.1.2. Comparisons of diversities and relative abundances of bacteria were conducted using a multivariate logistic regression. The FDR (Benjamini Hochberg) procedure was used for multiple test correction. The results were summarized as the estimated parameter, their standard errors and their p-values. UniFrac distances were compared using a non-parametric test (Nemenyi's test) for multiple comparisons test.

**Predictors of pneumonic infiltrate at exacerbation**

Clustered multivariate logistic modeling was chosen, since it adequately addresses the problem of repeated (clustered) information by subject, and it takes into account the fact that the dependent variable (presence or not of infiltrate in exacerbation) is dichotomous. Variables included in the regression model were: age, sex, body mass index, Global Initiative for Chronic Obstructive Lung Disease (GOLD) stage, season of exacerbation onset, cold/sore-throat symptoms, fever, sputum purulence, CRP, smoking status, eosinophil blood % at exacerbation (>2%), inhaled corticosteroid use, maintenance bronchodilator therapy, pneumococcal and influenza vaccination in the 12 months preceding enrolment.

**Reference**

(1) Bourne S, Cohet C, Kim V, et al. Acute Exacerbation and Respiratory InfectionS in COPD (AERIS): protocol for a prospective, observational cohort study. *BMJ Open.* 2014;4(3):e004546.
