# Supplementary material for: Impact of radiologically stratified exacerbations: insights into pneumonia aetiology in COPD
Source: Respir Res. 2018 Jul 28;19:143. doi: 10.1186/s12931-018-0842-8 (PMC6064093; doi:10.1186/s12931-018-0842-8)
Supplement: Supplementary file 2 — Table S1. Radiological findings at exacerbation. Table S2. Exacerbation treatment stratified by the presence or absence of radiographic pneumonic infiltrate. Table S3. Bacterial identification by culture and PCR in all exacerbation sputum samples and those exacerbation sputum samples with fewer than 30% squamous cells (considered high quality). Table S4. Bacterial and viral identification at exacerbation by culture (bacteria) and PCR (bacteria/viral). Table S5. Levels of inflammatory markers at paired stable and exacerbation visits. Table S6. Changes in levels of serum inflammatory markers between stable (pre-exacerbation) and exacerbation samples. Table S7. Levels of serum inflammatory markers at paired stable and exacerbation visits. The occurrence of the first infiltrate-associated exacerbation where available was prioritised, or first non-infiltrative exacerbation if not (subjects are therefore only represented once). Table S8. Lung function changes between nearest stable-state and exacerbation visits, stratified by the presence/absence of pneumonic infiltrate. Figure S1. The proportion of bacterial positive sputum samples at exacerbation by both culture and PCR. Figure S2. The lung microbiome (phylum) of exacerbations stratified by the presence or absence of pneumonic infiltrate. Figure S3. Area under the receiver operator curve analysis for CRP, fibrinogen and neutrophil count. (DOCX 232 kb) [file 12931_2018_842_MOESM2_ESM.docx]

**Table S1.** Radiological findings at exacerbation.

|  | All exacerbations (n=343) | Exacerbations without infiltrate (n=274) | Exacerbations with infiltrate (n=69) |
| --- | --- | --- | --- |
| Pleural effusion | 7 (2) | 5 (1.8) | 2 (2.9) |
| Cardiomegaly | 16 (4.7)* | 12 (4.4) | 5 (5.8) |
| Mass/nodule | 1 (0.3) | 1 (0.4) | 0 (0.0) |
| Pulmonary oedema | 1 (0.3) | 1 (0.4) | 0 (0.0) |

*7 CXRs at exacerbation with reported cardiomegaly are from one individual subject.

Data presented as n, (%)

**Table S2.** Exacerbation treatment stratified by the presence or absence of radiographic pneumonic infiltrate.

|  | Exacerbations without infiltrate  (n=274) | Exacerbations with infiltrate  (n=69) |
| --- | --- | --- |
| No treatment | 25 (9.1) | 5 (7.2) |
| Antibiotic only | 22 (8.0) | 11 (15.9) |
| OCS only | 62 (22.6) | 10 (14.5) |
| Antibiotics and OCS | 161 (58.8) | 43 (62.3) |
| Data unavailable | 4 (1.5) | 0 (0.0) |

Data presented as n, (%).

**Table S3.** Bacterial identification by culture and PCR in all exacerbation sputum samples and those exacerbation sputum samples with fewer than 30% squamous cells (considered high quality).

|  | All exacerbations with valid sputum | | | Only exacerbations with high quality sputum | | |
| --- | --- | --- | --- | --- | --- | --- |
|  | Infiltrate | No infiltrate | P-value | Infiltrate | No infiltrate | P-value |
| Culture | n=308 | | | n=209 | | |
| Any bacteria | 44 (69.8%) | 140 (57.1%) | 0.067 | 31 (70.5%) | 97 (58.8%) | 0.158 |
| *Haemophilus influenzae* | 33 (52.4%) | 93 (38.0%) | 0.038 | 25 (56.8%) | 67 (40.6%) | 0.054 |
| PCR | n=295 | | | n=204 | | |
| Any bacteria | 47 (77.0%) | 153 (65.4%) | 0.082 | 34 (77.3%) | 106 (66.3%) | 0.163 |
| *Haemophilus influenzae* | 38 (62.3%) | 122 (52.4%) | 0.165 | 26 (59.1%) | 85 (53.1%) | 0.482 |

Data represented as n (%). P-values from Chi-square test unless otherwise stated.

**Table S4.** Bacterial and viral identification at exacerbation by culture (bacteria) and PCR (bacteria/virus).

|  | Exacerbations with infiltrate | | Exacerbations without infiltrate | |  |
| --- | --- | --- | --- | --- | --- |
|  | n^♯^ |  | n^♯^ |  | P-Value |
| By culture | | | | | |
| Any bacteria | 63 | 44 (69.8) | 245 | 140 (57.1) | 0.067 |
| *Haemophilus influenzae* | 63 | 33 (52.4) | 245 | 93 (38.0) | 0.038 |
| *Streptococcus pneumoniae* | 63 | 7 (11.1) | 245 | 40 (16.3) | 0.305 |
| By PCR | | | | | |
| Any bacteria | 61 | 47 (77.0) | 233 | 153 (65.4) | 0.092 |
| *Haemophilus influenzae* | 61 | 38 (62.3) | 233 | 122 (52.4) | 0.165 |
| *Streptococcus pneumoniae* | 61 | 7 (11.5) | 233 | 19 (8.2) | 0.416 |
| Rhinovirus | 60 | 14 (23.3) | 234 | 56 (23.9) | 0.923 |
| Any virus | 60 | 25 (41.7) | 234 | 101 (43.2) | 0.835 |

Data represented as n (%). P-values from Chi-square test unless otherwise stated.

^♯^ indicates the number of subjects with available data/samples.

Any bacteria includes: *Haemophilus influenzae*, *Moraxella catarrhalis*, *Streptococcus pneumoniae*, *Pseudomonas aeruginosa*, and *Staphylococcus aureus*.

Any virus includes: influenza A, influenza B, respiratory syncytial virus, human metapneumovirus, parainfluenza virus 1–4, coronavirus (OC43, 229E, NL63, HKU1), rhinovirus/enterovirus, adenovirus, and bocavirus.

**Table S5.** Levels of serum inflammatory markers at paired stable, and exacerbation visits.

| Serum marker | Exacerbation with infiltrate | | | | Exacerbation without infiltrate | | | | P-value | P-value |
| --- | --- | --- | --- | --- | --- | --- | --- | --- | --- | --- |
|  | n^♯^ | Pre- AE stable visit | AE | P-value* | n^♯^ | Pre- AE stable visit | AE | P- value* | Stable- stable  ** | AE-AE  ** |
| CRP (mg/L) | 29 | 5.0 (11.0) | 19 (58.0) | <0.001 | 153 | 4.0 (6.0) | 7.0 (13.5) | <0.001 | 0.254 | <0.001 |
| Fibrinogen (g/L) | 29 | 4.8 (1.8) | 5.8 (2.3) | <0.001 | 137 | 4.6 (1.1) | 5.0 (1.5) | <0.001 | 0.868 | 0.005 |
| Blood WCC (cellsx10^9^L) | 31 | 7.8 (2.8) | 10.2 (4.5) | <0.001 | 150 | 7.6 (2.6) | 8.0 (3.5) | 0.010 | 0.518 | <0.001 |
| Blood neutrophils (cellsx10^9^L) | 31 | 5.1 (2.7) | 7.7 (4.4) | <0.001 | 150 | 4.8 (2.0) | 5.3 (3.2) | <0.001 | 0.414 | <0.001 |
| Procalcitonin (μg/L) | 32 | 0.0633 (0.0400) | 0.0798 (0.0400) | <0.001 | 151 | 0.0662 (0.0300) | 0.0723 (0.0400) | <0.001 | 0.187 | 0.220 |

AE: Acute exacerbation.

Data presented as median (IQR). * P-value from Wilcoxon signed rank test. ** P-values from Mann Whitney U test.

^♯^ indicates the number of subjects with available data/samples.

Blood was sampled at both stable visits and at exacerbation (within 72 hours of symptom onset) and results compared.

**Table S6.** Changes in levels of serum inflammatory markers between stable (pre-exacerbation) and exacerbation samples.

| Serum marker | Exacerbation with infiltrate | | Exacerbation without infiltrate | | P-value |
| --- | --- | --- | --- | --- | --- |
|  | n^♯^ |  | n^♯^ |  |  |
| Blood WCC (cellsx10^9^L) | 31 | 1.6 (3.8) | 150 | 0.4 (2.1) | 0.001 |
| Blood neutrophils (cellsx10^9^L) | 31 | 1.7 (3.7) | 150 | 0.5 (1.9) | 0.003 |
| CRP (mg/L) | 29 | 10.0 (59) | 153 | 2.0 (9.0) | 0.002 |
| Fibrinogen (g/L) | 29 | 0.9 (2.3) | 137 | 0.2 (1.2) | 0.009 |
| Procalcitonin (μg/L) | 32 | 0.019 (0.030) | 151 | 0.005 (0.030) | 0.002 |

Data presented as median (IQR). P-value from Mann Whitney U test.

^♯^ indicates the number of subjects with available data/samples.

Blood was sampled at both stable visits and at exacerbation (within 72 hours of symptom onset) and the median changes compared.

**Table S7.** Levels of serum inflammatory markers at paired stable, and exacerbation visits. The occurrence of the first infiltrate-associated exacerbation with available data was prioritised, or the first non-infiltrative exacerbation if not (subjects are therefore only represented once).

| Serum Marker | Exacerbations with infiltrate | | | | Exacerbations without infiltrate | | | | P-value | |
| --- | --- | --- | --- | --- | --- | --- | --- | --- | --- | --- |
|  | n^♯^ | Stable | AE | P-value* | n^♯^ | Stable | AE | P-value* | Stable-Stable** | AE-AE** |
| Blood WCC (cellsx10^9^L) | 27 | 7.6 (1.9) | 9.2 (5.5) | <0.001 | 73 | 7.5 (2.4) | 7.9 (3.0) | 0.031 | 0.810 | 0.001 |
| Blood neutrophils (cellsx10^9^L) | 27 | 4.5 (2.3) | 6.8 (4.7) | <0.001 | 73 | 4.7 (1.8) | 5.2 (2.5) | 0.026 | 0.652 | 0.002 |
| CRP (mg/L) | 25 | 5.0 (10.5) | 19.0 (56.5) | 0.001 | 76 | 4.0 (6.0) | 8.0 (13.0) | <0.001 | 0.202 | 0.001 |
| Fibrinogen (g/L) | 25 | 4.8 (1.7) | 5.8 (2.2) | 0.001 | 64 | 4.8 (1.0) | 5.3 (1.5) | <0.001 | 0.798 | 0.019 |
| Procalcitonin (μg/L) | 28 | 0.0651 (0.0400) | 0.0759 (0.0400) | 0.001 | 74 | 0.0652 (0.0300) | 0.0686 (0.0400) | 0.172 | 0.409 | 0.117 |

Stable = visit prior to exacerbation in which patient deemed to be at stable-state; AE = acute exacerbation visit.

Data presented as median (IQR). *P-value from Wilcoxon ranked sign test. **P-value from Mann Whitney U test.

^♯^ indicates the number of subjects with available data/samples.

Blood was sampled at both stable visits and at exacerbation (within 72 hours of symptom onset) and results compared.

**Table S8.** Lung function changes between nearest stable-state and exacerbation visits, stratified by presence/absence of pneumonic infiltrate.

|  | Exacerbations with infiltrate  (n=36) | Exacerbations without infiltrate  (n=203) | P-value |
| --- | --- | --- | --- |
| FEV1 (ml) | -125 (0.18) | -40 (0.17) | 0.010 |
| FVC (ml) | -180 (0.49) | -70 (0.39) | 0.091 |

FVC=forced vital capacity; FEV1=forced expiratory volume in 1 second.

Data presented as median (IQR). P-value from Mann Whitney U test.

**Figure S1.** The proportion of bacterial positive sputum samples at exacerbation, by both culture and PCR. ‘Any bacteria’ comprise *Haemophilus influenzae*, *Streptococcus pneumoniae*, *Moraxella catarrhalis*, *Pseudomonas aeruginosa* and *Staphylococcus aureus*.


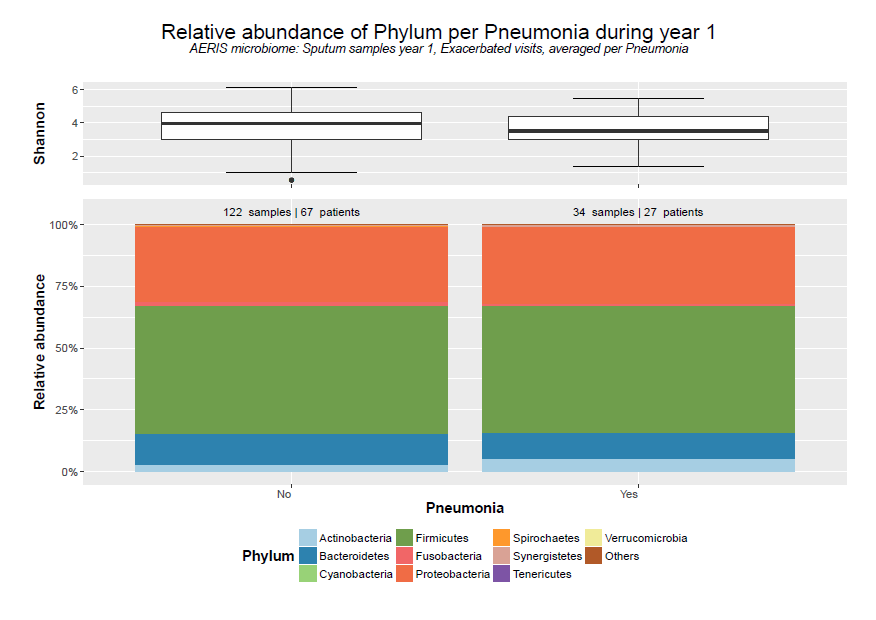


**Figure S2.** The lung microbiome of exacerbations, stratified by the presence or absence of pneumonic infiltrate. (A) The Shannon diversity index did not show any significant difference between radiological groups (p=0.45). (B) The relative phylum abundances showed no significant differences between groups (p=0.35).

**
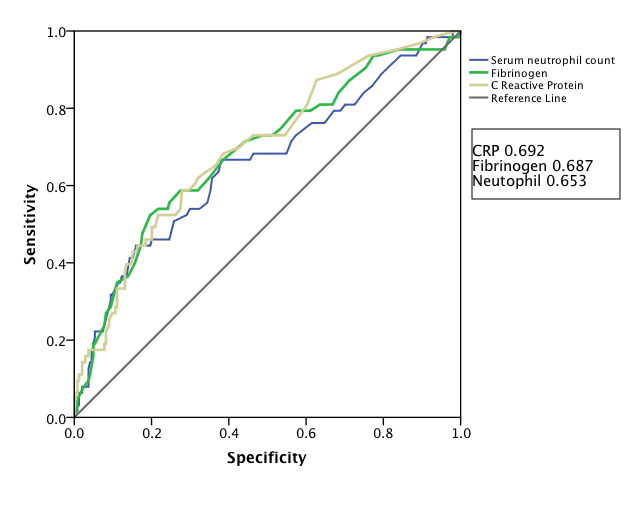
**

**Figure S3.** Area under the receiver operator curve analysis for CRP, fibrinogen and neutrophil count.
